# Bis(2,4,6-trimethyl­phen­yl)zinc(II)

**DOI:** 10.1107/S1600536809023186

**Published:** 2009-06-20

**Authors:** Sven Krieck, Helmar Görls, Matthias Westerhausen

**Affiliations:** a Institute of Inorganic and Analytical Chemistry, Friedrich-Schiller-Universität Jena, August-Bebel-Strasse 2, D-07743 Jena, Germany

## Abstract

The title compound, [Zn(C_9_H_11_)_2_] or Mes_2_Zn (Mes =  mesityl = 2,4,6-trimethyl­phen­yl), crystallizes with a quarter of a mol­ecule in the asymmetric unit. The Zn^II^ atom is in a strictly linear environment with a Zn—C bond length of 1.951 (5) Å. Due to the imposed 2/*m * symmetry, both aromatic rings are coplanar. One of the methyl groups is disordered over two equally occupied positions.

## Related literature

For the first synthesis of dimesitylzinc, see: Seidel & Bürger (1981 ▶). For related structures, see: Brooker *et al.* (1992 ▶); Cole *et al.* (2003 ▶); Markies *et al.* (1990 ▶); Sun *et al.* (1998 ▶); Weidenbruch *et al.* (1989 ▶); Westerhausen *et al.* (2005 ▶).
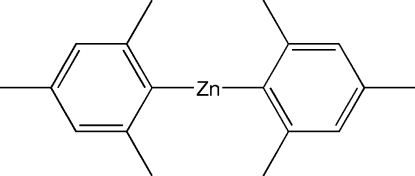


## Experimental

### 

#### Crystal data

[Zn(C_9_H_11_)_2_]
*M* *_r_* = 303.73
Tetragonal, 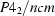

*a* = 18.3059 (9) Å
*c* = 5.0494 (4) Å
*V* = 1692.08 (18) Å^3^
*Z* = 4
Mo *K*α radiation
μ = 1.44 mm^−1^
*T* = 183 K
0.05 × 0.05 × 0.04 mm

#### Data collection

Nonius KappaCCD diffractometer
Absorption correction: none
10286 measured reflections
1016 independent reflections
685 reflections with *I* > 2σ(*I*)
*R*_int_ = 0.046

#### Refinement

*R*[*F* ^2^ > 2σ(*F* ^2^)] = 0.071
*wR*(*F* ^2^) = 0.270
*S* = 1.13
1016 reflections
53 parameters
H-atom parameters constrained
Δρ_max_ = 1.33 e Å^−3^
Δρ_min_ = −0.60 e Å^−3^

### 

Data collection: *COLLECT* (Nonius, 1998 ▶); cell refinement: *DENZO* (Otwinowski & Minor, 1997 ▶); data reduction: *DENZO*; program(s) used to solve structure: *SHELXS97* (Sheldrick, 2008 ▶); program(s) used to refine structure: *SHELXL97* (Sheldrick, 2008 ▶); molecular graphics: *SHELXTL/PC* (Sheldrick, 2008 ▶); software used to prepare material for publication: *SHELXL97*.

## Supplementary Material

Crystal structure: contains datablocks I, global. DOI: 10.1107/S1600536809023186/bt2971sup1.cif

Structure factors: contains datablocks I. DOI: 10.1107/S1600536809023186/bt2971Isup2.hkl

Additional supplementary materials:  crystallographic information; 3D view; checkCIF report

## Figures and Tables

**Fig. 1.**
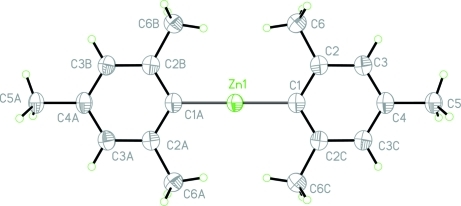
Molecular structure of Mes2Zn, showing 40% probability displacement ellipsoides and the atom numbering scheme.

## supplementary crystallographic information

### Comment

After the first synthesis of dimesitylzinc by Seidel & Bürger (1981), its structure was determined more than 20 years later (Cole *et al.*, 2003). Here we present another modification of this diarylzinc compound.

Whereas dialkylzinc is monomeric diphenylzinc crystallizes as a loose and unsymmetric dimer (Markies *et al.* (1990)). A planar molecule with a strictly two coordinated zinc centre is observed for bis(2,4,6-trimethylphenyl)zinc (dimesitylzinc) by Cole *et al.* (2003). Other substitution patterns of the arene ring also lead to monomeric, but not strictly linear molecules in the solid state. Sun *et al.* (1998) published the structure of bis(pentafluorophenyl)zinc and Brooker *et al.* (1992) reported the structure of bis[2,4,6-tris(rifluoromethyl)phenyl]zinc with a C—Zn—C bond angle of 170°. A The C—Zn—C angle decreases with increasing steric chain and a value of 165.9° was found in bis[2,4,6-tri(*tert* -butylphenyl]zinc by Westerhausen *et al.* (2005).

### Experimental

All manipulations were performed in an atmosphere of argon using standard Schlenk techniques. THF and toluene were dried (Na/benzophenone) and distilled prior to use. Mes_2_Zn was prepared according to a literature procedure (Seidel & Bürger, 1981). Recrystallization of Mes_2_Zn from toluene at +4%C led to the formation of single crystals of the title compound.

### Refinement

All hydrogen atoms were set to idealized positions and were refined with 1.2 times (1.5 for methyl groups) the isotropic displacement parameter of the corresponding carbon atom. One of the methyl groups is disordered over two equally occupied positions. The structure contains solvent accessible voids. But the final difference peak of 1.33 e/A^3^ is on a special position and could not be related to a solvent molecule.

### Crystal data

**Table:**

[Zn(C_9_H_11_)_2_]	*D*_x_ = 1.192 Mg m^−^^3^
*M**_r_* = 303.73	Mo *K*α radiation, λ = 0.71073 Å
Tetragonal, *P*4_2_/*n**c**m*	Cell parameters from 10286 reflections
Hall symbol: -P 4ac 2ac	θ = 3.2–27.5°
*a* = 18.3059 (9) Å	µ = 1.44 mm^−^^1^
*c* = 5.0494 (4) Å	*T* = 183 K
*V* = 1692.08 (18) Å^3^	Octaeder, colourless
*Z* = 4	0.05 × 0.05 × 0.04 mm
*F*(000) = 640	

### Data collection

**Table:**

Nonius KappaCCD diffractometer	685 reflections with *I* > 2σ(*I*)
Radiation source: fine-focus sealed tube	*R*_int_ = 0.046
graphite	θ_max_ = 27.5°, θ_min_ = 3.2°
φ and ω scans	*h* = −22→23
10286 measured reflections	*k* = −23→23
1016 independent reflections	*l* = −6→5

### Refinement

**Table:**

Refinement on *F*^2^	Secondary atom site location: difference Fourier map
Least-squares matrix: full	Hydrogen site location: inferred from neighbouring sites
*R*[*F*^2^ > 2σ(*F*^2^)] = 0.071	H-atom parameters constrained
*wR*(*F*^2^) = 0.270	*w* = 1/[σ^2^(*F*_o_^2^) + (0.1807*P*)^2^ + 0.5133*P*] where *P* = (*F*_o_^2^ + 2*F*_c_^2^)/3
*S* = 1.13	(Δ/σ)_max_ < 0.001
1016 reflections	Δρ_max_ = 1.33 e Å^−^^3^
53 parameters	Δρ_min_ = −0.60 e Å^−^^3^
0 restraints	Extinction correction: *SHELXL97* (Sheldrick, 2008), Fc^*^=kFc[1+0.001xFc^2^λ^3^/sin(2θ)]^-1/4^
Primary atom site location: structure-invariant direct methods	Extinction coefficient: 0.041 (11)

### Special details

**Table:**

Geometry. All e.s.d.'s (except the e.s.d. in the dihedral angle between two l.s. planes) are estimated using the full covariance matrix. The cell e.s.d.'s are taken into account individually in the estimation of e.s.d.'s in distances, angles and torsion angles; correlations between e.s.d.'s in cell parameters are only used when they are defined by crystal symmetry. An approximate (isotropic) treatment of cell e.s.d.'s is used for estimating e.s.d.'s involving l.s. planes.
Refinement. Refinement of *F*^2^ against ALL reflections. The weighted *R*-factor *wR* and goodness of fit *S* are based on *F*^2^, conventional *R*-factors *R* are based on *F*, with *F* set to zero for negative *F*^2^. The threshold expression of *F*^2^ > σ(*F*^2^) is used only for calculating *R*-factors(gt) *etc*. and is not relevant to the choice of reflections for refinement. *R*-factors based on *F*^2^ are statistically about twice as large as those based on *F*, and *R*- factors based on ALL data will be even larger.

### Fractional atomic coordinates and isotropic or equivalent isotropic displacement parameters (Å^2^)

**Table:**

	*x*	*y*	*z*	*U*_iso_*/*U*_eq_	Occ. (<1)
Zn1	0.0000	0.5000	0.0000	0.0388 (6)	
C1	−0.0524 (2)	0.5524 (2)	−0.2778 (9)	0.0397 (13)	
C2	−0.1176 (2)	0.5250 (2)	−0.3816 (7)	0.0413 (11)	
C3	−0.1542 (2)	0.5623 (2)	−0.5831 (8)	0.0424 (11)	
H3A	−0.1987	0.5431	−0.6500	0.051*	
C4	−0.1268 (2)	0.6268 (2)	−0.6875 (9)	0.0432 (14)	
C5	−0.1687 (2)	0.6687 (2)	−0.8986 (11)	0.0455 (14)	
H5A	−0.1418	0.7132	−0.9452	0.068*	0.50
H5B	−0.2170	0.6820	−0.8302	0.068*	0.50
H5C	−0.1743	0.6381	−1.0563	0.068*	0.50
C6	−0.1516 (2)	0.4566 (2)	−0.2699 (8)	0.0503 (12)	
H6A	−0.1933	0.4422	−0.3799	0.075*	
H6B	−0.1683	0.4659	−0.0886	0.075*	
H6C	−0.1153	0.4172	−0.2687	0.075*	

### Atomic displacement parameters (Å^2^)

**Table:**

	*U*^11^	*U*^22^	*U*^33^	*U*^12^	*U*^13^	*U*^23^
Zn1	0.0385 (7)	0.0385 (7)	0.0394 (9)	0.0034 (3)	−0.0015 (2)	0.0015 (2)
C1	0.0435 (19)	0.0435 (19)	0.032 (2)	0.008 (2)	0.0036 (14)	−0.0036 (14)
C2	0.043 (2)	0.044 (2)	0.038 (2)	0.0059 (17)	0.0021 (16)	−0.0033 (16)
C3	0.046 (2)	0.045 (2)	0.0353 (19)	0.0052 (16)	0.0002 (16)	−0.0052 (17)
C4	0.051 (2)	0.051 (2)	0.028 (2)	0.010 (3)	0.0028 (14)	−0.0028 (14)
C5	0.054 (2)	0.054 (2)	0.029 (3)	0.005 (3)	−0.0019 (16)	0.0019 (16)
C6	0.052 (3)	0.045 (2)	0.054 (2)	−0.0005 (18)	−0.0011 (19)	0.0024 (19)

### Geometric parameters (Å, °)

**Table:**

Zn1—C1	1.951 (5)	C4—C3^ii^	1.386 (5)
Zn1—C1^i^	1.951 (5)	C4—C5	1.522 (7)
C1—C2^ii^	1.396 (5)	C5—H5A	0.9800
C1—C2	1.396 (5)	C5—H5B	0.9800
C2—C3	1.397 (6)	C5—H5C	0.9800
C2—C6	1.509 (6)	C6—H6A	0.9800
C3—C4	1.386 (5)	C6—H6B	0.9800
C3—H3A	0.9500	C6—H6C	0.9800
			
C1—Zn1—C1^i^	179.999 (1)	C4—C5—H5A	109.5
C2^ii^—C1—C2	118.1 (5)	C4—C5—H5B	109.5
C2^ii^—C1—Zn1	120.9 (2)	H5A—C5—H5B	109.5
C2—C1—Zn1	120.9 (2)	C4—C5—H5C	109.5
C1—C2—C3	120.6 (4)	H5A—C5—H5C	109.5
C1—C2—C6	120.7 (4)	H5B—C5—H5C	109.5
C3—C2—C6	118.7 (3)	C2—C6—H6A	109.5
C4—C3—C2	121.3 (4)	C2—C6—H6B	109.5
C4—C3—H3A	119.4	H6A—C6—H6B	109.5
C2—C3—H3A	119.4	C2—C6—H6C	109.5
C3^ii^—C4—C3	118.1 (5)	H6A—C6—H6C	109.5
C3^ii^—C4—C5	120.9 (2)	H6B—C6—H6C	109.5
C3—C4—C5	120.9 (2)		

Symmetry codes: (i) −*x*, −*y*+1, −*z*; (ii) −*y*+1/2, −*x*+1/2, *z*.
